# Oligodendrogliomas, IDH-mutant and 1p/19q-codeleted, arising during teenage years often lack *TERT* promoter mutation that is typical of their adult counterparts

**DOI:** 10.1186/s40478-018-0598-x

**Published:** 2018-09-19

**Authors:** Julieann Lee, Angelica R. Putnam, Samuel H. Chesier, Anuradha Banerjee, Corey Raffel, Jessica Van Ziffle, Courtney Onodera, James P. Grenert, Boris C. Bastian, Arie Perry, David A. Solomon

**Affiliations:** 1 Department of Pathology, University of California, San Francisco, CA USA; 2 Department of Pathology, University of Utah School of Medicine, Salt Lake City, UT USA; 3 Department of Neurosurgery, University of Utah School of Medicine, Salt Lake City, UT USA; 4 Division of Hematology/Oncology, Department of Pediatrics, University of California, San Francisco, CA USA; 5 Department of Neurological Surgery, University of California, San Francisco, CA USA; 6 Clinical Cancer Genomics Laboratory, University of California, San Francisco, CA USA

**Keywords:** Oligodendroglioma, IDH mutation, 1p/19q-codeletion, Teenager, Pediatric, *TERT* promoter, *FGFR1*, *CIC*, *IDH1*, Molecular neuro-oncology

The genetic alterations that characterize oligodendroglial neoplasms have been defined over the past decade. In adults, oligodendrogliomas are genetically defined by the combination of *IDH1* p.R132 or *IDH2* p.R172 mutation, *TERT* promoter hotspot mutation (either c.-124C > T or c.-126C > T), and chromosomes 1p and 19q co-deletion, which is frequently accompanied by mutations involving *CIC*, *FUBP1*, *TCF12*, *NOTCH1*, and *PIK3CA* genes [2, 3, 7, 13, 21]. Oligodendrogliomas in children often lack the IDH mutation, *TERT* promoter mutation, and 1p/19q-codeletion that is observed in their adult counterparts [14, 20]. Instead, they most commonly harbor solitary pathogenic alterations in the *FGFR1* oncogene that cause constitutive activation of the kinase domain via gene fusion, tandem duplication, or missense mutations that localize at one of two hotspots (p.N546 or p.K656) [18, 23].

To investigate the molecular pathogenesis of oligodendrogliomas arising during teenage years, we assembled a cohort of tissue specimens from three patients (Fig. 1a). The one male and two female patients ranged in age at time of initial surgery from 10-18 years. All patients presented with headaches that led to brain imaging, which demonstrated non-enhancing, T2-hyperintense masses centered in the frontal (n=2) or parietal (n=1) lobes (Fig. 1b). All cases were histologically characterized by an infiltrative glial neoplasm composed of cells with uniform round nuclei containing delicate chromatin (Fig. 1c). Mitoses were inconspicuous, and neither microvascular proliferation nor necrosis were present. Immunohistochemistry revealed that the tumor cells were OLIG2 positive, had intact/retained nuclear expression of ATRX protein, and showed only occasional positivity for p53 protein. The Ki-67 labeling index was uniformly low (less than 2%).

Genomic DNA was extracted from formalin-fixed, paraffin-embedded tumor tissue, and targeted capture-based next-generation DNA sequencing was performed as previously described using the UCSF500 Cancer Panel [8, 9, 11, 16, 17], which assesses approximately 500 cancer-associated genes for mutations, copy number alterations, and structural variants including gene fusions (Additional file 1: Table S1). All three cases demonstrated IDH mutation, with two harboring *IDH2* p.R172K and one harboring *IDH1* p.R132H (Additional file 1: Table S2 and Additional file 2: Fig. S1). Additionally, case #1 contained a damaging missense mutation in *TP53* (p.R175H), which was present at a subclonal allele frequency (6%) relative to the *IDH1* mutation (20%), indicating that it was only present in a subset of tumor cells. Case #3 additionally contained a truncating nonsense mutation in the *CIC* tumor suppressor gene (p.S349*). No pathogenic mutations were identified involving any of the other genes targeted for sequencing by this assay. Chromosomal copy number analysis revealed losses of 1p and 19q in all three cases, which uniformly involved the entire arms of these chromosomes. No other chromosomal gains, losses, or focal amplifications or deletions were identified in any of the tumors (Additional file 1: Table S3 and Additional file 2: Fig. S2). Notably, all three cases lacked mutation at either of the two hotspots in the promoter region of the *TERT* gene (Fig. 1d), and also did not harbor either *TERT* gene amplification or structural rearrangement within the 50 Kb of upstream sequence covered by this assay, where rearrangements are commonly found in high-risk neuroblastomas, chromophobe renal cell carcinomas, and IDH-wildtype glioblastomas lacking *TERT* promoter hotspot mutation [5, 6, 15, 22].

**Fig. 1:**
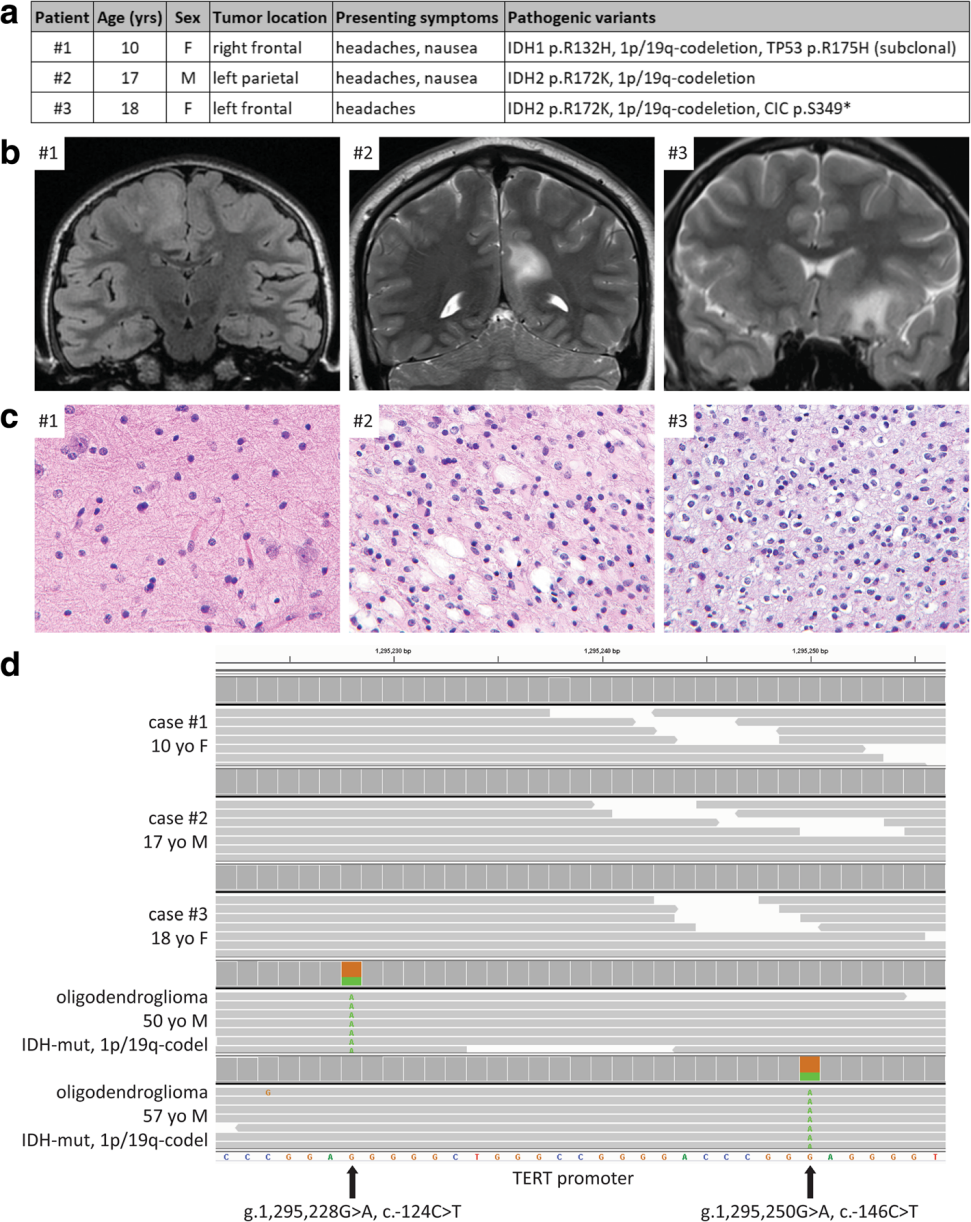
Oligodendrogliomas, IDH-mutant and 1p/19q-codeleted, arising during teenage years often lack *TERT* promoter hotspot mutation. **a**, Table of the clinicopathologic features of the three teenagers with oligodendroglioma. **b**, Pre-operative magnetic resonance imaging for the three patients. **c**, Histology of the three tumors. Hematoxylin and eosin staining, 60× magnification. **d**, Snapshot from the Integrated Genome Viewer for the three oligodendrogliomas in teenagers demonstrating absence of the *TERT* promoter hotspot mutation seen in oligodendrogliomas, IDH-mutant and 1p/19q-codeleted, from adults

In order to further assess the frequency of *TERT* promoter mutation in IDH-mutant and 1p/19q-codeleted oligodendrogliomas in teenagers, we examined data from the four major pediatric low-grade glioma genomics studies published to date [1, 18, 19, 23]. Together, these four studies included only a single teenage patient with an oligodendroglioma, IDH-mutant and 1p/19q-codeleted, in which *TERT* promoter status had been assessed. This patient (SJLGG034 from the Zhang et al study, also labeled LGNT20 in the Qaddoumi et al study) was a 15 year old male with an oligodendroglioma that harbored *IDH1* p.R132H mutation, multiple *CIC* mutations, 1p/19q-codeletion, and reportedly lacked *TERT* promoter mutation [18, 23]. We next examined data from the most recent glioma metagenomics study by The Cancer Genome Atlas Research Network that included 89 cases of oligodendroglioma, IDH-mutant and 1p/19q-codeleted, WHO grade II or III, in which *TERT* promoter status was reported [4]. 87 of these 89 cases (98%) reportedly harbored *TERT* promoter hotspot mutation and were all in adults (age range 20-75 years at diagnosis). Two of the 89 cases are reported to be *TERT* promoter wildtype, one in a teenager and one in an older adult. The first *TERT* promoter wildtype oligodendroglioma case (TCGA-DB-5278) was centered in the left frontal lobe of a 17 year old male who had presented with seizures, demonstrated WHO grade II histologic features, *IDH1* p.R132H mutation, *CIC* mutation, 1p/19q-codeletion, and did not have *TERT* overexpression. The second *TERT* promoter wildtype oligodendroglioma case (TCGA-HT-8010) was in a 64 year old female, had WHO grade II histologic features, *IDH* mutation, *NF1* mutation, 1p/19q-codeletion, and also did not have *TERT* overexpression. Thus, according to the latest published dataset from The Cancer Genome Atlas, *TERT* promoter mutation was present in 87/88 cases (99%) of IDH-mutant and 1p/19q-codeleted oligodendrogliomas in adults age 20+ years at time of diagnosis. In contrast, *TERT* promoter mutation was present in 0/5 cases (0%) of IDH-mutant and 1p/19-codeleted oligodendrogliomas in teenagers, including the three patients from our cohort, one patient from Zhang et al, and one patient from The Cancer Genome Atlas.

Together, these findings suggest that oligodendrogliomas arising during teenage years are genetically distinct from their adult counterparts based on the absence of *TERT* promoter mutation. Though telomere maintenance has been proposed as a requirement for gliomagenesis in adults [10, 12], it does not appear to be necessary in oligodendrogliomas, IDH-mutant and 1p/19q-codeleted, in teenagers. We speculate that this may be due to the low number of cell divisions that have taken place in oligodendrogliomas arising in teenagers relative to adults such that selection pressure for acquisition of telomere maintenance mechanism has not yet occurred. The absence of telomerase activation in these tumors may potentially correlate with the less frequent anaplasia and more indolent clinical behavior that has been observed in pediatric oligodendrogliomas compared to their adult counterparts [20].

## Additional files

Additional file 1: **Table S1.** List of the 479 genes targeted for sequencing on the UCSF500 Cancer Panel. **Table S2.** Pathogenic mutations identified in the three oligodendrogliomas. (XLSX 20 kb)

Additional file 2: **Figure S1.** Snapshots from the Integrated Genome Viewer of the IDH mutations present in each of the three oligodendrogliomas, IDH-mutant and 1p/19q-codeleted, in teenagers. **Fig. S2.** Chromosomal copy number and zygosity plots for the oligodendroglioma, IDHmutant and 1p/19q-codeleted, from teenage patient #2. (PDF 950 kb)
