# Supplementary material for: Oligodendrogliomas, IDH-mutant and 1p/19q-codeleted, arising during teenage years often lack TERT promoter mutation that is typical of their adult counterparts
Source: Acta Neuropathol Commun. 2018 Sep 19;6:95. doi: 10.1186/s40478-018-0598-x (PMC6145350; doi:10.1186/s40478-018-0598-x)
Supplement: Supplementary file 2 — Figure S1. Snapshots from the Integrated Genome Viewer of the IDH mutations present in each of the three oligodendrogliomas, IDH-mutant and 1p/19q-codeleted, in teenagers. Fig. S2. Chromosomal copy number and zygosity plots for the oligodendroglioma, IDHmutant and 1p/19q-codeleted, from teenage patient #2. (PDF 950 kb) [file 40478_2018_598_MOESM2_ESM.pdf]

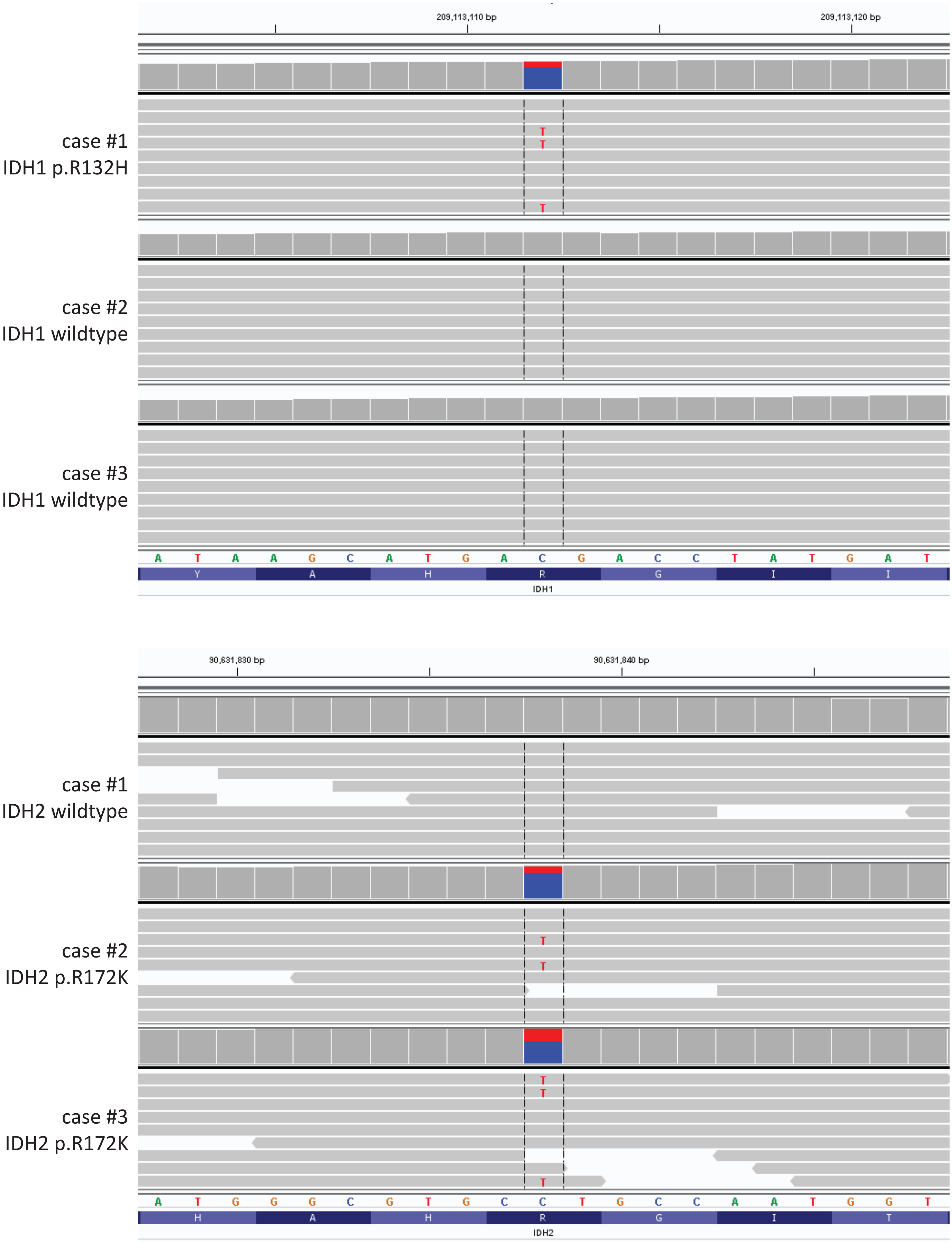

Supplementary Figure 1. Snapshots from the Integrated Genome Viewer of the IDH mutations present in each of the three oligodendrogliomas, IDH-mutant and 1p/19q-codeleted, in teenagers.

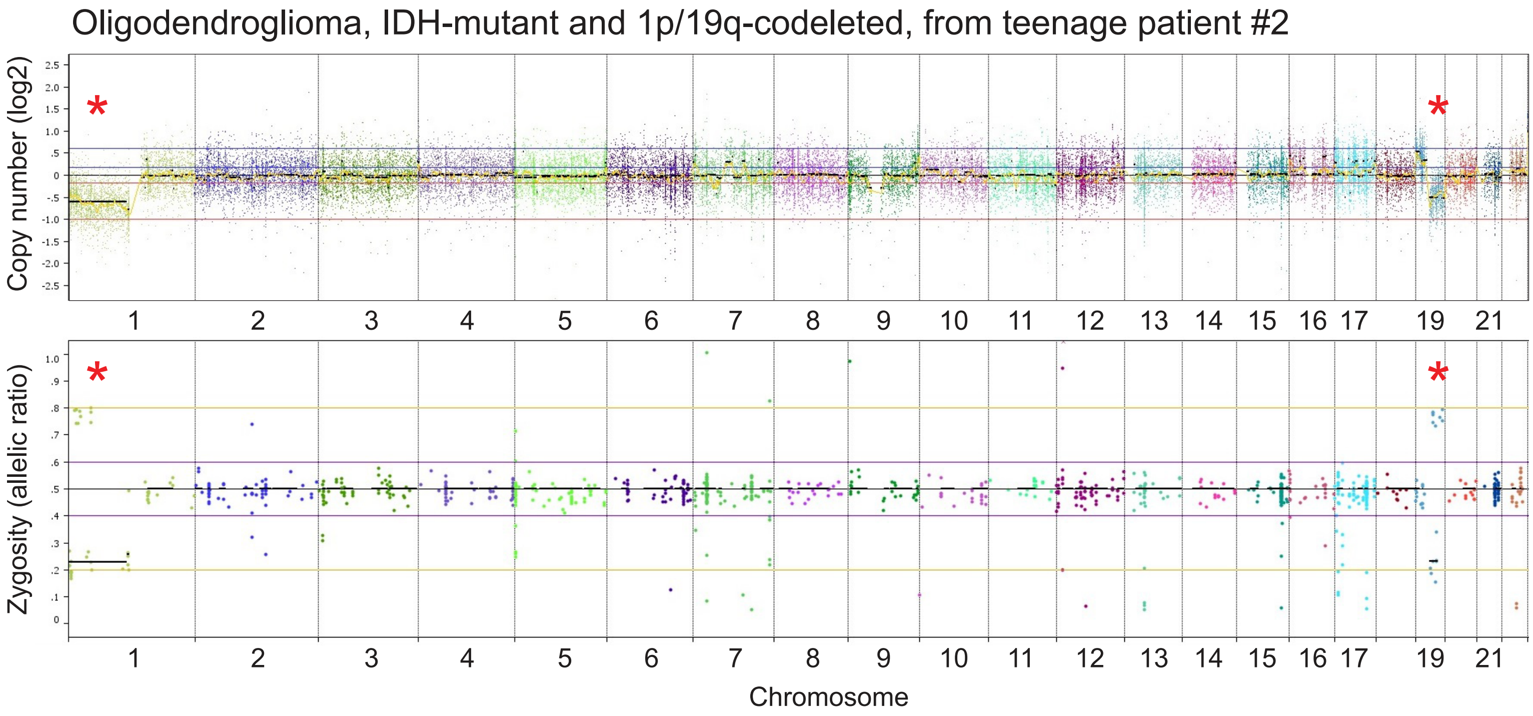

Supplementary Figure 2. Chromosomal copy number and zygosity plots for the oligodendroglioma, IDH-mutant and 1p/19q-codeleted, from teenage patient #2. Losses of the entire arms of chromosomes 1p and 19q are the solitary copy number changes present.
